# QuickStats

**Published:** 2015-05-01

**Authors:** FROM THE NATIONAL CENTER FOR HEALTH STATISTICS

**Figure:**
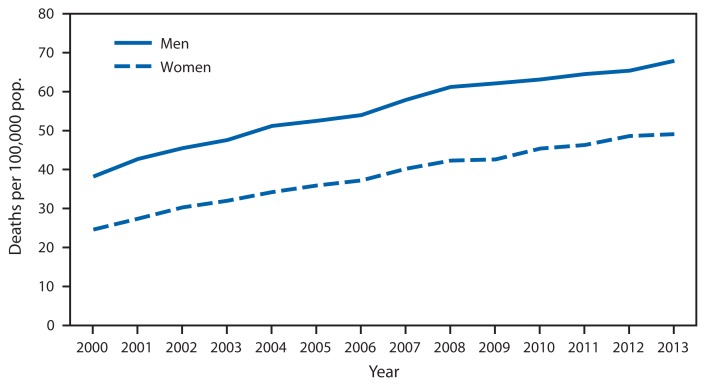
Death Rates* from Unintentional Falls^†^ Among Adults Aged ≥65 Years, by Sex — United States, 2000–2013 * Rates are age-adjusted using the 2000 U.S. standard population. ^†^ Deaths from unintentional falls are identified using International Classification of Diseases, Tenth Revision (ICD-10) underlying cause of death codes W00–W19. There were 10,273 deaths in 2000 and 25,464 in 2013 from unintentional falls among adults aged ≥65.

During 2000–2013, age-adjusted death rates from unintentional falls increased steadily for both men and women aged ≥65 years, with consistently higher rates observed among men. During this period, death rates from falls increased from 38.2 per 100,000 population in 2000 to 67.9 in 2013 among men and from 24.6 to 49.1 among women.

**Source:** National Vital Statistics System mortality data. Available at http://www.cdc.gov/nchs/deaths.htm.

**Reported by:** Yahtyng Sheu, PhD, ysheu@cdc.gov, 301-458-4354, Li-Hui Chen, PhD, Holly Hedegaard, MD, MSPH.

